# Identification of Kinase Targets for Enhancing the Antitumor Activity of Eribulin in Triple-Negative Breast Cell Lines

**DOI:** 10.3390/biomedicines11030735

**Published:** 2023-02-28

**Authors:** Xuemei Xie, Jangsoon Lee, Jon A. Fuson, Huey Liu, Toshiaki Iwase, Kyuson Yun, Cori Margain, Debu Tripathy, Naoto T. Ueno

**Affiliations:** 1 Section of Translational Breast Cancer Research, Department of Breast Medical Oncology, The University of Texas MD Anderson Cancer Center, Houston, TX 77030, USA; 2 Research Institute at Houston Methodist, Weill Cornell Medical College, Houston, TX 77030, USA; 3 Empiri Inc., Houston, TX 77054, USA; 4 Cancer Biology and Therapeutics, University of Hawai’i Cancer Center, Honolulu, HI 96813, USA

**Keywords:** triple-negative breast cancer, eribulin, everolimus, copanlisib, the PI3K/mTOR pathway

## Abstract

Background: Triple-negative breast cancer (TNBC) is the most aggressive molecular subtype of breast cancer, and current treatments are only partially effective in disease control. More effective combination approaches are needed to improve the survival of TNBC patients. Eribulin mesylate, a non-taxane microtubule dynamics inhibitor, is approved by the U.S. Food and Drug Administration to treat metastatic breast cancer after at least two previous chemotherapeutic regimens. However, eribulin as a single agent has limited therapeutic efficacy against TNBC. Methods: High-throughput kinome library RNAi screening, Ingenuity Pathway Analysis, and STRING analysis were performed to identify target kinases for combination with eribulin. The identified combinations were validated using in vivo and ex vivo proliferation assays. Results: We identified 135 potential kinase targets whose inhibition enhanced the antiproliferation effect of eribulin in TNBC cells, with the PI3K/Akt/mTOR and the MAPK/JNK pathways emerging as the top candidates. Indeed, copanlisib (pan-class I PI3K inhibitor), everolimus (mTOR inhibitor), trametinib (MEK inhibitor), and JNK-IN-8 (pan-JNK inhibitor) produced strong synergistic antiproliferative effects when combined with eribulin, and the PI3K and mTOR inhibitors had the most potent effects in vitro. Conclusions: Our data suggest a new strategy of combining eribulin with PI3K or mTOR inhibitors to treat TNBC.

## 1. Introduction

Triple-negative breast cancer (TNBC), which lacks the expression of estrogen receptor (ER) and progesterone receptor (PR) and has low or no expression of human epidermal growth factor receptor 2 (HER2), accounts for 15–20% of all breast cancers diagnosed worldwide [1]. Compared with patients with other subtypes of breast cancer, patients with TNBC have shorter survival; the 5-year mortality rate of patients with TNBC is 40% [2]. TNBC is more aggressive than other subtypes of breast cancer and has a worse prognosis [3,4,5]. The poor outcomes of TNBC are attributed to its high rates of metastasis and relapse [3,4,5]. In addition, previous clinical trials have shown the limited efficacy of several small-molecule inhibitors and monoclonal antibodies against tumor-driving signaling pathways in TNBC [6,7,8]. The current U.S. Food and Drug Administration (FDA)-approved targeted drugs for TNBC treatment are poly(ADP-ribose) polymerase (PARP) inhibitors, an anti-PD-L1 immune checkpoint inhibitor, and an antibody-drug conjugate targeting trophoblast cell-surface antigen 2 (TROP2). However, the use of these drugs in patients with TNBC is limited because only some TNBC patients are eligible for these treatments, and resistance ultimately develops. As a reslult, cytotoxic systemic chemotherapy still has a significant role in TNBC treatment.

Another vulnerability in TNBC may lie in the PI3K/Akt/mTOR pathway, that plays a critical role in cell proliferation, survival, mobility, and chemotherapy resistance in breast cancer [9]. This pathway can be highly activated by multiple genomic alterations, including mutations in the oncogenes *PIK3CA*, *AKT*, and *MTOR* and inactivating mutations in the tumor suppressor genes *PIK3R1*, *INPP4B*, *PTEN*, *TSC1*, *TSC2*, and *LKB1* (also called *STK11*) [10]. *PIK3CA,* an oncogene that encodes the p110α catalytic subunit of phosphatidylinositol-3-kinase (PI3K), is the second most commonly mutated gene after *P53* in TNBC [11]. Mutations in *PIK3CA* can activate several signaling pathways in breast cancer, predominantly affecting the PI3K/Akt pathway [12,13]. *PIK3CA* mutations increase TNBC cell aggressiveness and confer resistance to chemotherapy in TNBC by inducing constitutive activation of PI3K/Akt/mTOR signaling and suppressing apoptosis [14]. This association with chemotherapy resistance is further supported by the association of *PIK3CA* mutations with a poor pathological complete response rate to neoadjuvant chemotherapy in TNBC [15,16]. *PIK3CA* mutations are detected in 37% of ER+/HER2−, 22% of HER2+, and 18% of ER−/HER2− breast tumors and in 23.7% of TNBC [17,18]. The higher rate of *PIK3CA* mutations in advanced TNBC relative to early-stage TNBC is likely due to ER expression loss in relapsed breast cancer that was initially positive for ER, a subtype that harbors a high rate of *PIK3CA* mutations [19]. For all these reasons, inhibitors targeting the PI3Kα subunit of *PIK3CA* have been tested in TNBC.

In the same pathway, mTOR is another possible therapeutic target for TNBC. mTOR, a serine–threonine kinase, forms two complexes, mTORC1 and mTORC2, and is mutated in 1.8% of the primary breast cancer cases in *The Cancer Genome Atlas*, with a small minority of these mutations recognized as putative drivers [20,21]. In addition to genetic activation, mTOR can be activated by PI3K signaling, which has been associated with drug resistance, and mTOR inhibition re-sensitizes ER+ breast cancer cells to tamoxifen [22]. The FDA has approved the mTOR inhibitor everolimus for the treatment of advanced HER2− breast cancer that expresses either ER or PR.

A treatment that has shown activity in TNBC is eribulin (eribulin mesylate). Eribulin is a synthetic analog of halichondrin B, a natural product isolated from the marine sponge *Halichondria okadai* [23]. It functions as a non-taxane microtubule inhibitor [24]. The FDA has approved eribulin to treat patients with advanced or metastatic breast cancer who have received at least two chemotherapy regimens. Eribulin exerts its antitumor activity by binding to microtubule ends to prevent microtubule polymerization, leading to cell cycle arrest at the G2/M phase and subsequent apoptosis [24]. In addition, eribulin inhibits tumor progression or metastasis by reducing hypoxia [25], reversing epithelial–mesenchymal transition [26], suppressing migration and invasion [26], and preventing tumor vasculature remodeling [25]. Eribulin has been used to treat advanced or metastatic breast cancer, including TNBC [27,28,29], and has been used to overcome chemotherapy resistance. Eribulin produced regression of cisplatin-resistant tumors in a patient-derived xenograft model of triple-negative matrix-producing breast carcinoma [30]. The EMBRACE trial, a phase III study of eribulin in patients with HER2− advanced breast cancer, showed overall survival improvement after eribulin treatment in patients whose breast cancer was resistant to anthracycline-based regimens and taxane therapy [31]. However, the improvement of overall survival in eribulin-treated patients was only 2.5 months compared with the treatment of physician’s choice, and clinical resistance to eribulin developed after a median of 3.7 months [31].

To further enhance the anti-tumor efficacy of eribulin against TNBC, we aimed to identify potential synergistic partners for eribulin through a high-throughput kinome library RNA interference (RNAi) screening. We found that the PI3K/Akt/mTOR, MAPK, and JNK pathways are potential targets for eribulin-based combination treatment. We confirmed that targeting PI3K with copanlisib (a pan-class I PI3K inhibitor) and targeting mTOR with everolimus showed synergistic effects when combined with eribulin. Our data indicate that either of these kinase inhibitors combined with eribulin represent a promising combination therapy for TNBC.

## 2. Materials and Methods

### 2.1. Cell Lines and Reagents

BT-20, MDA-MB-157, MDA-MB-453, MDA-MB-468, MDA-MB-231, HCC70, HCC1187, HCC1806, BT-549, HCC1395, and MDA-MB-436 TNBC cells and MCF10A normal breast epithelial cells were purchased from American Type Culture Collection (Manassas, VA, USA). HCC2185 and HCC3153 TNBC cells were purchased from The University of Texas Southwestern Medical Center (Dallas, TX, USA). MFM-223, CAL51, and CAL120 TNBC cells were purchased from the DSMZ-German Collection of Microorganisms and Cell Cultures (Braunschweig, Germany). SUM159 (TNBC), SUM185 (TNBC), and SUM149 (triple-negative inflammatory breast cancer (TN-IBC)) cells were purchased from Asterand Bioscience (Detroit, MI, USA). The BCX-010 (TN-IBC) cell line was established at The University of Texas MD Anderson Cancer Center (Houston, TX, USA).

BT-20, HCC70, HCC1187, HCC1806, BT-549, HCC1395, HCC2185, and HCC3153 (TNBC) cells were maintained in Roswell Park Memorial Institute 1640 medium (#R8758; Sigma-Aldrich, St. Louis, MO, USA). MDA-MB-157, MDA-MB-453, MDA-MB-468, MDA-MB-231, and MDA-MB-436 (TNBC) cells were maintained in Dulbecco’s modified Eagle medium/F-12 medium (#D8062; Sigma-Aldrich). BCX-010, SUM149, and SUM159 (TN-IBC) cells were maintained in Ham’s F-12 medium (#11765054; Life Technologies Inc., Carlsbad, CA, USA) supplemented with 5 µg/mL insulin (#12-585-014; Thermo Fisher Scientific Inc., Waltham, MA, USA) and 1 µg/mL hydrocortisone (#H0888; Sigma-Aldrich). All media were supplemented with 10% fetal bovine serum (FBS; #F0600-050; GenDEPOT, Katy, TX, USA) and 1% antibiotic/antimycotic (#A5955; Sigma-Aldrich). MCF10A cells were maintained in Dulbecco’s modified Eagle medium/F-12 medium, supplemented with 5% horse serum (#16050-122; Thermo Fisher Scientific), 5 µg/mL insulin, 1 µg/mL hydrocortisone, 1% antibiotic/antimycotic, 100 ng/mL cholera toxin (#C8052; Sigma-Aldrich), and 20 ng/mL epidermal growth factor (#HZ-1326; Proteintech Group, Inc., Rosemont, IL, USA). All cell lines were validated by DNA typing at the MD Anderson Cancer Center Characterized Cell Line Core and confirmed to be free of mycoplasma using the MycoAlert Mycoplasma Detection Kit (#LT07-710; Lonza, Morristown, NJ, USA).

### 2.2. Small Molecules

Copanlisib (#204570) was purchased from MedKoo (Morrisville, NC, USA). Trametinib (#S2673), everolimus (#S1120) and JNK-IN-8 (#S4901) were purchased from Selleckchem (Houston, TX, USA). Eribulin was provided by Eisai Inc. (Nutley, NJ, USA).

### 2.3. Cell Proliferation Assay

The anti-proliferation effect of eribulin against TNBC and IBC cells was assessed using CellTiter-Blue Cell viability and sulforhodamine B staining assays. In brief, 3 to 6 × 10^3^ cells/well were seeded in 96-well plates and treated the next day with eribulin alone or with eribulin combined with kinase inhibitors, including copanlisib, trametinib, everolimus, and JNK-IN-8, for 5 days. On day 5, following treatments, the CellTiter-Blue reagent (#PR-G8081; Promega Corporation, Madison, WI, USA) was added to the plates, and the optical density (595 nm) was determined using a Victor X3 plate reader (PerkinElmer, Waltham, MA, USA). The cells were then fixed with 5% trichloroacetic acid (#T8657; Sigma-Aldrich) for 2 h at room temperature and then stained with 0.03% sulforhodamine B solution (#230162; Sigma-Aldrich) for 30 min at room temperature. The stained cells were dissolved in 10 mM Tris buffer (#1610732; Bio-Rad, Hercules, CA, USA). The optical density was determined at 585 nm using the Victor X3 plate reader. Growth inhibition graphs were generated, and IC_50_ values were calculated using nonlinear regression to fit the data to the log (inhibitor) vs. response (variable slope) curve using GraphPad Prism software 9 (GraphPad Software, Inc., San Diego, CA, USA). To evaluate the synergistic anti-proliferation effects of eribulin in combination with kinase inhibitors, the combination index (CI) and fraction affected were determined using the CalcuSyn software (v2.1, Biosoft, Cambridge, United Kingdom). CI < 0.90 indicates a synergistic impact, 0.91 ≤ CI < 1.10 indicates an additive impact, and CI ≥ 1.11 indicates an antagonistic impact of the two-drug combination.

### 2.4. Soft Agar Colony Formation Assay

The impact of drug treatment on anchorage-independent growth was assessed using a soft agar assay, a standard method to predict in vivo carcinogenesis and a drug’s anti-tumor efficacy. Cells (2 to 5 × 10^3^ cells/well) were suspended in 2 mL of 0.375% agarose with DMSO or drugs and then overlaid onto 650 µL of 0.75% agarose layer in six-well plates. The plates were incubated for 3 to 6 weeks. After treatment, the colonies were stained with 200 µL of MTT (3-(4,5-dimethylthiazol-2-yl)-2,5-diphenyltetrazolium bromide; 2 mg/mL; #M5655; Sigma-Aldrich) for 2 h. Stained colonies greater than 80 μm in diameter were counted using a GelCount system (Oxford Optronix Ltd., Milton, UK).

### 2.5. High-Throughput RNAi Screening

Pooled kinome siRNA, consisting of 3 unique siRNAs targeting 1 gene and a total of 2127 siRNAs targeting 709 kinase genes, was selected using Ambion Silencer Select Human Genome siRNA Library V4 (#4397926; Life Technologies Inc.) and placed into Greiner Bio-One Cellstar 384-well plates (Thermo Fisher Scientific). Seventy-five microliters of the three pooled siRNAs (2 µM) for one gene were dispensed per well in quadruplicate. For an internal control, Silencer Select Negative Control No. 1 siRNA (#4390844; Thermo Fisher Scientific), Silencer Select Positive Control PLK1 siRNA (#AM51331; Thermo Fisher Scientific), and no-siRNA control (Thermo Fisher Scientific) were included in each plate. The positive control (PLK1 siRNA) was used to assess transfection efficiency, and the no-siRNA control was used to calculate cellular sensitivity to eribulin. Lipofectamine RNAiMAX (0.05 µL/well; #13778030; Thermo Fisher Scientific) in 10 µL of serum-free Opti-MEM was added to the plates, and then the plates were incubated at room temperature for 45 min. After incubation, SUM149 (300 cells) in 20 µL of complete media without antibiotics was added. The plates were sealed and incubated at 37 °C with 5% CO_2_ for 48 h. A total of 28 plates were prepared for kinome screening, with each plate containing pooled siRNAs targeting 26 genes. At 48 h after treatment with pooled siRNAs, eribulin at the final concentration of IC_20_ (0.3 nM) or vehicle (DMSO) in 30 µL of complete media was added to each well. The plates were then sealed and incubated at 37 °C with 5% CO_2_ for 72 h. Following drug treatment, 35 µL of medium was aspirated from each well, followed by the addition of ATPlite 1step (25 µL/well; #6016731; PerkinElmer). The plates were sealed and incubated at room temperature for 10 min by shaking at 1000 rpm on an orbital shaker. Luminescence readings were used as an indicator of cell viability. Luminescence readings for each treatment were averaged and normalized to the mean of the DMSO-treated no-siRNA negative control in the same plate as relative viability. The median Z’-factor in each plate was calculated using the equation below. The median z’-factor was 0.58 for the SUM149 kinome screening.

Z’factor = 1 − [3 × (σ_p_ + σ_n_)]/(μ_p_ − μ_n_)

Here, σ_p_ is the standard deviation of the PLK1 positive control, σ_n_ is the standard deviation of the no-siRNA negative control, μ_p_ is the mean of the PLK1 positive control, and μ_n_ is the mean of the no-siRNA negative control.

To assess the impact of siRNA-induced gene knockdown on drug sensitivity, the sensitivity index was calculated for each gene after eribulin treatment to identify synergistic drug and gene combination hits, using the equation below [32]. Each drug and gene combination was then ranked according to this sensitivity index score with a cutoff of 0.15.

Sensitivity index = (Rc/Cc × Cd/Cc) − Rd/Cc

Here, Rc is the viability of cells treated with siRNA without the drug, Cc is the viability of cells treated with the no-siRNA no-drug control, Cd is the viability of cells treated with the drug, and Rd is the viability of cells treated with siRNA plus drug.

### 2.6. Western Blotting

Cells (3 × 10^5^ cells/10 mL) were seeded in 6-cm plates overnight and were treated the next day with eribulin alone or in combination with everolimus, trametinib, JNK-IN-8, or copanlisib for 48 h at 37 °C. At 48 h following treatment, the total protein was extracted using a cold M-PER Mammalian Protein Extraction Reagent (#78501; Thermo Fisher Scientific) complemented with phosphatase and protease inhibitors (#B15001; Bimake.com, Houston, TX, USA). The protein samples were denatured by incubation at 70 °C for 10 min on a heat plate, resolved by running sodium dodecyl sulfate–polyacrylamide gel electrophoresis using NuPAGE 4–12% Bis-Tris Plus gel (#WG1403BOX, Thermo Fisher Scientific), and then transferred onto a polyvinylidene difluoride membrane (#1620177, Bio-Rad). Following blocking with 4% bovine serum albumin, proteins of interest on the blots were probed using the following primary antibodies (in a 1:1000 dilution) purchased from Cell Signaling Technology (Danvers, MA, USA) or other suppliers as indicated: anti-XIAP (#14334), anti-phospho-AKT (#4060), anti-phospho-mTOR (#5536), anti-phospho-H2AX (#80312), anti-PARP (#9542), or anti-α-tubulin (#T9026, Sigma-Aldrich). The secondary antibodies used were horseradish peroxidase–conjugated immunoglobulin G (Life Technologies Inc.) for chemiluminescence signal detection. The intensity of target proteins on the blots was measured using ImageJ (National Institutes of Health, Bethesda, MD, USA).

### 2.7. Caspase 3/7 Activity Measurement

Caspase 3/7 activity was measured using Caspase-Glo 3/7 assay reagent (#G8091; Promega Corporation) according to the manufacturer’s instructions. In brief, cells (5 × 10^3^ cells) were seeded into 96-well plates and cultured overnight. The next morning, the cells were treated with eribulin alone, everolimus alone, copanlisib alone, eribulin plus everolimus, or eribulin plus copanlisib for 24 h at 37 °C. At 24 h after treatment, the culture media were removed, and 100 µL of the Caspase-Glo 3/7 assay reagent was added into the plates, which were incubated for 1 h at 37 °C. Luminescence intensity was determined using the Victor X3 plate reader (PerkinElmer), and the background signal from the control wells containing reagent only was subtracted.

### 2.8. Cell Cycle Analysis

Cells (3 × 10^5^ cells) were seeded into 60-mm plates and cultured overnight. The next morning, the cells were treated with eribulin alone, everolimus alone, copanlisib alone, eribulin plus everolimus, or eribulin plus copanlisib for 72 h at 37 °C. At 72 h after treatment, the cells were collected, fixed in ice-cold 70% EtOH (#111000200; Greenfield Global Inc., Toronto, ON, Canada), treated with 50 μg/mL RNase A (#12091021; Invitrogen), stained with propidium iodide (#P4864; Sigma-Aldrich), and subjected to flow cytometry analysis.

### 2.9. Cell Migration Assay

The migration assay was performed using a 24-well microchemotaxis chamber (Corning Inc., Corning, NY, USA). Cells (2 × 10^6^) were seeded in 10-cm plates and the next morning treated with eribulin alone, everolimus alone, copanlisib alone, eribulin plus everolimus, or eribulin plus copanlisib for 2 h at 37 °C. At 2 h after treatment, the cells were resuspended in FBS-free medium (2.5 × 10^5^ cells/250 µL) and then added into the upper chambers of transwells separated by inserts with 8-µm pores. The lower chambers were filled with complete medium (750 µL) containing 10% FBS as an attractant. The cells were allowed to migrate for 6-8 h and then fixed and stained with hematoxylin and eosin. Migrated cells were scanned using the PathScan Enabler IV Pathology Slide Scanner (Meyer Instruments, Inc., Houston, TX, USA) and then quantified using National Institutes of Health ImageJ software (http://rsb.info.nih.gov/ij/ (accessed on 17 January 2023)).

### 2.10. Ex Vivo Determination of Drug Sensitivity

Fresh tissue slices (300 µm) were prepared from TNBC cell xenografts collected from humanized NSG-SGM3 mice (Jackson Laboratory, Bar Harbor, ME, USA) and cultured in an air/media interface on a membrane. Every 4 days, the media were discarded, and fresh media containing drugs were added. Viability was measured on day 8 or 12 using the luminescent viability assay.

### 2.11. Statistical Analysis

The cell proliferation rate was summarized with descriptive statistics (mean, median, and quartiles) and box plots for each treatment group. A two-tailed unpaired Student *t*-test was used for statistical analysis using GraphPad Prism software, with *p* ≤ 0.05 considered significant.

## 3. Results

### 3.1. Eribulin Suppressed Proliferation of TNBC and TN-IBC Cells

**Figure 1:**
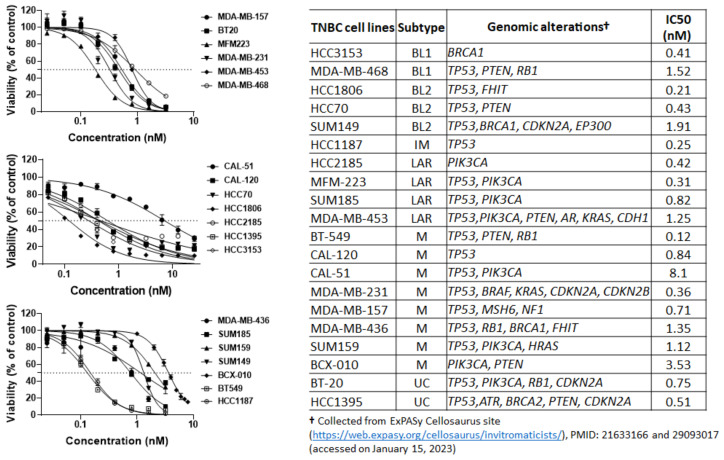
Anti-proliferation activity of eribulin against TNBC and IBC cells. Cells were seeded in 96-well plates and treated the next day with eribulin for 5 days. On day 5 following treatments, the cells were fixed with 5% trichloroacetic acid and then stained with 0.025% sulforhodamine B solution. The stained cells were dissolved in 10 mM Tris buffer. The optical density at 585 nm was measured using a Victor X3 plate reader. The data are representative of three independent experiments with three replications for each experiment. Cell viability is presented as a percentage of non-treated control cells. Data are presented as mean ± standard deviation. BL1, basal-like 1; BL2, basal-like 2; IM, immunomodulatory; LAR, luminal androgen receptor; M, mesenchymal; UC, unclassified.

To evaluate the antitumor efficacy of eribulin in vitro and to select proper cell lines for the further evaluation of eribulin’s antitumor efficacy in combinations ex vivo, we tested eribulin for its anti-proliferation effects against a panel of TNBC and IBC cells. As shown by a sulforhodamine B assay, eribulin effectively inhibited the proliferation of the 18 tested TNBC cell lines, which were BT-20, MFM-223, MDA-MB-157, MDA-MB-453, MDA-MB-468, MDA-MB-231, HCC70, HCC2185, CAL-120, HCC1187, CAL-51, HCC1806, SUM185, BT-549, HCC1395, HCC3153, MDA-MB-436, and SUM159 (Figure 1). Eribulin also effectively inhibited the proliferation of the two tested TN-IBC cell lines, which were SUM149 and BCX-010 (Figure 1). The IC_50_ values of eribulin against the tested TNBC and IBC cells ranged from 0.12 nM to 1.91 nM, except for CAL51, for which the IC_50_ was 8.1 nM. These results demonstrate that eribulin is highly effective at inhibiting the proliferation of both TNBC and TN-IBC cells, with IC_50_ values in the nanomolar range.

Despite this overall effectiveness, we observed variation in the TNBC cell lines’ responses to eribulin (Figure 1). These varying responses may be related to the differences in TNBC molecular subtypes and genomic alterations in the PI3K/Akt/mTOR pathway. TNBC is a heterogeneous disease with different molecular subtypes showing different responses to chemotherapy [33]. In addition, the mutations in *MTOR*, *PIK3CA*, *TP53*, *PTEN*, and/or other genes have a significant impact on responses to chemotherapy [9,14,15,16,22]. Indeed, based on TNBC molecular subtypes and genomic alterations in the PI3K/Akt/mTOR pathway, we noted that basal-like 1 (MDA-MB-468), basal-like 2 (SUM149), and luminal androgen receptor (MDA-MB-453) TNBC cells harboring more mutations in this pathway were less responsive to eribulin than the cells of the same subtypes harboring fewer mutations (Table in Figure 1). We also observed that, among the tested mesenchymal TNBC cell lines, the cells harboring mutations in *PIK3CA* and *TP53* (CAL-51, SUM159) or *PIK3CA* and *PTEN* (BCX-010) were less responsive to eribulin than the cells harboring other mutations. Altogether, our results demonstrate that different TNBC molecular subtypes with different mutations in the PI3K/Akt/mTOR pathway have different responses to eribulin. This should be considered in the development of eribulin-based combination treatments.

### 3.2. PI3K/Akt/mTOR and MAPK/JNK Pathways Potentiate the Antitumor Efficacy of Eribulin in TNBC Cells

**Figure 2:**
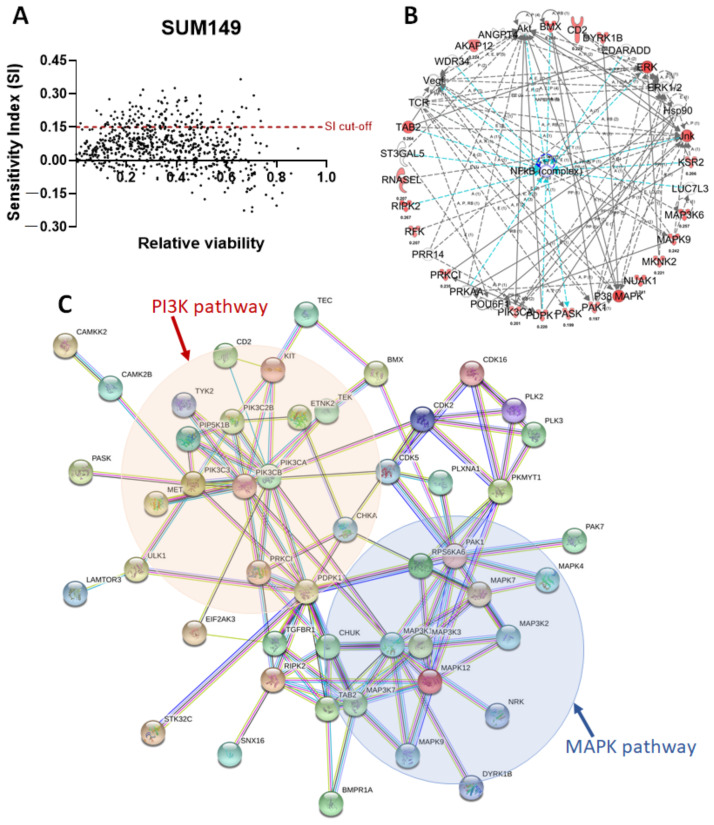
Network identified by the synthetic lethal high-throughput RNAi screening in SUM149 cells. (**A**) Synthetic lethal high-throughput RNAi screening was performed with a sensitivity index cutoff of 0.15 in SUM149 cells. (**B**) An ingenuity pathway analysis of pathways involving 135 potential target genes whose inhibition enhances the anti-proliferation effect of eribulin. (**C**) Protein–protein interaction analysis of target genes (STRING V11) identified the PI3K/Akt/mTOR and the MAPK/JNK pathways as the top candidates whose inhibition significantly enhances the anti-proliferation effect of eribulin against SUM149 cells.

To identify potential synergistic partners to improve the antitumor efficacy of eribulin against TNBC, we performed a high-throughput RNAi screening using kinome library pooled siRNA, consisting of 2127 siRNAs targeting 709 kinase genes, in SUM149 cells; these cells are well characterized in preclinical models, are chemoresistant, and are relatively resistant to eribulin (Table 1) [34]. Based on a sensitivity index with a cutoff at 0.15, we identified 135 genes whose inhibition significantly enhanced the anti-proliferation effect of eribulin against SUM149 cells (Table 1). Furthermore, an ingenuity pathway analysis identified the PI3K/Akt/mTOR and MAPK/JNK pathways as the top candidates whose inhibition using siRNA significantly enhanced the anti-proliferation effect of eribulin against SUM149 cells (Figure 2). These results suggest that targeting the PI3K/Akt/mTOR or MAPK/JNK pathway may improve the antitumor efficacy of eribulin in TNBC.

### 3.3. Combination with Inhibitors of Target Kinases Enhanced Anti-Proliferation Activity of Eribulin

To validate the potential of targeting the PI3K/Akt/mTOR and the MAPK/JNK pathways to enhance the antitumor efficacy of eribulin in TNBC, we tested the synergistic effect of eribulin and kinase inhibitors, including everolimus (an mTOR inhibitor), copanlisib (a pan–class I PI3K inhibitor), trametinib (a MEK inhibitor), and JNK-IN-8 (a pan–JNK inhibitor) using MDA-MB-468, MDA-MB-436, SUM149, and BCX-010 TNBC cells. These cell lines can easily form tumors in mice and have either functionally normal *PIK3CA* (MDA-MB-436 and SUM149) or mutant *PIK3CA* (MDA-MB-468 and BCX-010). Therefore, these cell lines allow us to understand the impact of *PIK3CA* mutations on the synergistic effect of eribulin combined with everolimus or copanlisib.

**Figure 3:**
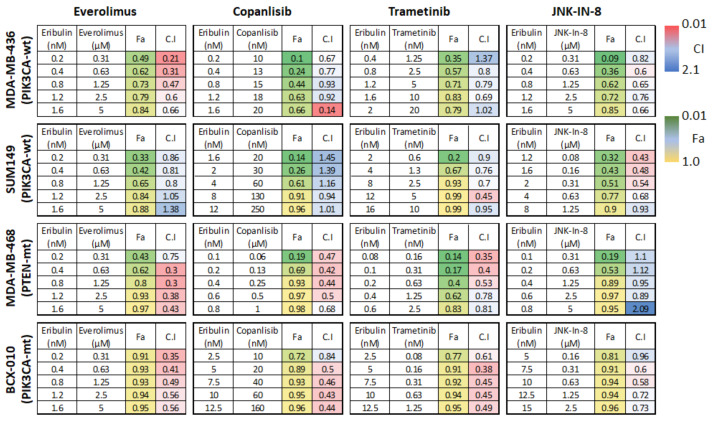
Fraction affected (Fa) and combination index (CI) of eribulin combined with other drugs in TNBC cell lines. CIs indicate the following: <0.1, very strong synergism; 0.10–0.30, strong synergism; 0.31–0.70, synergism; 0.71–0.85, moderate synergism; 0.86–0.90, slight synergism; 0.91–1.10, nearly additive; 1.11–1.20, slight antagonism; 1.21–1.45, moderate antagonism; 1.46–3.30, antagonism; 3.31–10, strong antagonism; >10, very strong antagonism. Fa 1.0 indicates 100% cell death. mt, mutated; wt, wild-type.

As shown in Figure 3, the combination of eribulin and everolimus showed a strong synergistic anti-proliferation effect in MDA-MB-468, MDA-MB-436, and BCX-010 cells, with CIs ranging from 0.21 to 0.75. This combination also showed a moderate synergistic effect at low doses and a slight antagonism effect at high doses in SUM149 cells, with CIs ranging from 0.80 to 1.38. The combination of eribulin and copanlisib showed a strong synergistic anti-proliferation effect in MDA-MB-468 and BCX-010 cells, with CIs ranging from 0.42 to 0.84. This combination also showed a moderate synergistic effect in MDA-MB-436 cells, with CIs ranging from 0.14 to 0.93. No synergistic effect of eribulin and copanlisib was observed in SUM149 cells, with CIs ranging from 0.94 to 1.45. The combination of eribulin and trametinib showed a synergistic anti-proliferation effect in MDA-MB-468, SUM149, and BCX-010 cells, with CIs ranging from 0.35 to 0.95, and a moderate synergistic to moderate antagonistic effect in MDA-MB-436 cells, with CIs ranging from 0.69 to 1.37. The combination of eribulin and JNK-IN-8 showed a synergistic to nearly additive anti-proliferation effect in MDA-MB-436, SUM149, and BCX-010 cells, with CIs ranging from 0.43 to 0.96, whereas no synergistic effect was observed in MDA-MB-468 cells, with CIs ranging from 0.89 to 2.09.

Altogether, the combination of eribulin with everolimus had the most synergistic anti-proliferation effect, especially in MDA-MB-468, MDA-MB-436, and BCX-010 cells, followed by the combinations of eribulin with trametinib or copanlisib, with JNK-IN-8 showing the least synergy. This study indicates that inhibition of mTOR/PI3K signaling by everolimus or by copanlisib may improve the anti-tumor efficacy of eribulin in TNBC. Among the tested cell lines, BCX-010 cells, followed by MDA-MB-468 cells, showed the most synergistic response to all the tested combination treatments.

### 3.4. Combination with Inhibitors of the PI3K/mTOR Pathway Enhanced Growth Inhibition Activity of Eribulin in 3D Culture

**Figure 4:**
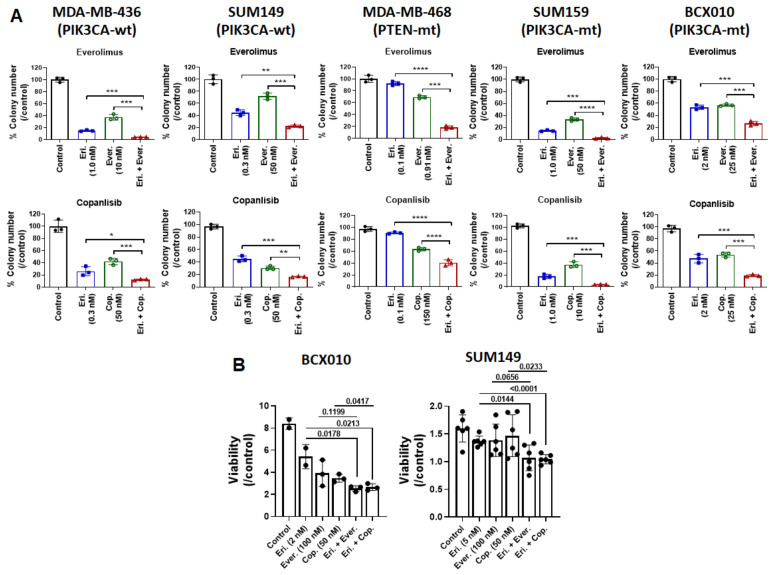
Combination of eribulin with everolimus or copanlisib is more effective than monotherapies at inhibiting TNBC cell growth. The synergistic effects of combination treatment were determined using (**A**) a soft agar colony formation assay at weeks 3 or 4 and (**B**) an ex vivo model following treatment with eribulin alone, everolimus alone, copanlisib alone, eribulin plus everolimus, or eribulin plus copanlisib. Data are presented as mean ± standard deviation. In (**A**), colony numbers are presented as percentage of non-treated control cells. *, *p* < 0.05; **, *p* < 0.01; ***, *p* < 0.001, ****, *p* < 0.0001 by two-tailed Student *t*-test. Eri., eribulin; Ever., everolimus; Cop., copanlisib; mt, mutated; wt, wild-type.

The results from the 2D culture suggest that, compared with the inhibition of other pathways, the inhibition of the mTOR/PI3K pathway showed the most synergistic effect when combined with eribulin. Therefore, we further tested the synergistic antitumor effect of eribulin combined with everolimus or copanlisib by performing an anchorage-independent soft agar colony formation assay, that reflects the in vivo tumorigenicity of tumor cells and in vivo antitumor efficacy of therapeutic agents. Meanwhile, to evaluate the impact of PIK3CA and PTEN mutations on the synergistic effect of eribulin with everolimus or copanlisib, we used cell lines harboring mutations in *PIK3CA* (SUM159 and BCX-010) or *PTEN* (MDA-MB-468). As shown in Figure 4A and Supplementary Figure S1, compared to eribulin or everolimus monotherapy, combination therapy with eribulin and everolimus significantly suppressed colony formation 5.97-fold vs. eribulin (*p* < 0.001) and 13.66-fold vs. everolimus (*p* < 0.001) in MDA-MB-436 cells; 2.89-fold vs. eribulin (*p* < 0.01) and 5.51-fold vs. everolimus (*p* < 0.001) in SUM149 cells; 5.05-fold vs. eribulin (*p* < 0.0001) and 3.79-fold vs. everolimus (*p* < 0.001) in MDA-MB-468 cells; 5.76-fold vs. eribulin (*p* < 0.001) and 12.96-fold vs. everolimus (*p* < 0.0001) in SUM159 cells; and 11.04-fold vs. eribulin (*p* < 0.001) and 1.82-fold vs. everolimus (*p* < 0.001) in BCX-010 cells. Similarly, compared to eribulin or copanlisib monotherapy, the combination of eribulin with copanlisib significantly suppressed tumors 2.12-fold vs. eribulin (*p* < 0.05) and 3.43-fold vs. copanlisib (*p* < 0.001) in MDA-MB-436 cells; 2.65-fold vs. eribulin (*p* < 0.001) and 4.53-fold vs. copanlisib (*p* < 0.01) in SUM149 cells; 2.44-fold vs. eribulin (*p* < 0.0001) and 1.69-fold vs. copanlisib (*p* < 0.0001) in MDA-MB-468 cells; 3.84-fold vs. eribulin (*p* < 0.001) and 9.54-fold vs. copanlisib (*p* < 0.001) in SUM159 cells; and 3.09-fold vs. eribulin (*p* < 0.001) and 3.05-fold vs. copanlisib (*p* < 0.001) in BCX-010 cells. Furthermore, to assess the potential toxicity of the combination treatment to non-tumorigenic cells, we also examined the effect of the treatment on the growth of normal breast epithelial cell line MCF10A. As shown in Supplementary Figure S2A, no synergistic effects were observed in MCF10A cells when eribulin was combined with everolimus or copanlisib, suggesting that the synergistic effect is tumor cell specific.

Next, we examined the synergistic anti-proliferation effect of eribulin combined with everolimus or copanlisib in BCX-010 and SUM149 cells using an ex vivo (patient-derived xenograft fresh tumor slice) model. As shown in Figure 4B, compared to eribulin or everolimus monotherapy, combination therapy with eribulin and everolimus reduced the viability of BCX-010 cells by 69.87% vs. eribulin (*p* = 0.0178) and by 53.47% vs. everolimus (*p* = 0.1199); the combination therapy also reduced the viability of SUM149 cells by 31.36% vs. eribulin (*p* = 0.0144) and by 11.10% vs. everolimus (*p* = 0.0656). Similarly, compared to eribulin or copanlisib monotherapy, the combination of eribulin with copanlisib significantly reduced the viability of BCX-010 cells by 68.41% vs. eribulin (*p* = 0.0213) and by 58.79% vs. copanlisib (*p* = 0.0417); the combination therapy also reduced the viability of SUM149 cells by 32.93% vs. eribulin (*p* < 0.0001) and by 5.38% vs. copanlisib (*p* = 0.0233).

These results clearly demonstrate that the inhibition of the PI3K/mTOR pathway effectively enhances the anti-tumor efficacy of eribulin in vitro. Moreover, the cell lines harboring *PTEN* or *PIK3CA* mutations showed a similar synergistic response to that of the wild-type cell lines, indicating that these mutations do not impact this synergistic effect.

### 3.5. Combination of Eribulin with Inhibitors of Target Kinases Arrested Cell Cycle Progression and Induced Apoptosis in TNBC Cells

**Figure 5:**
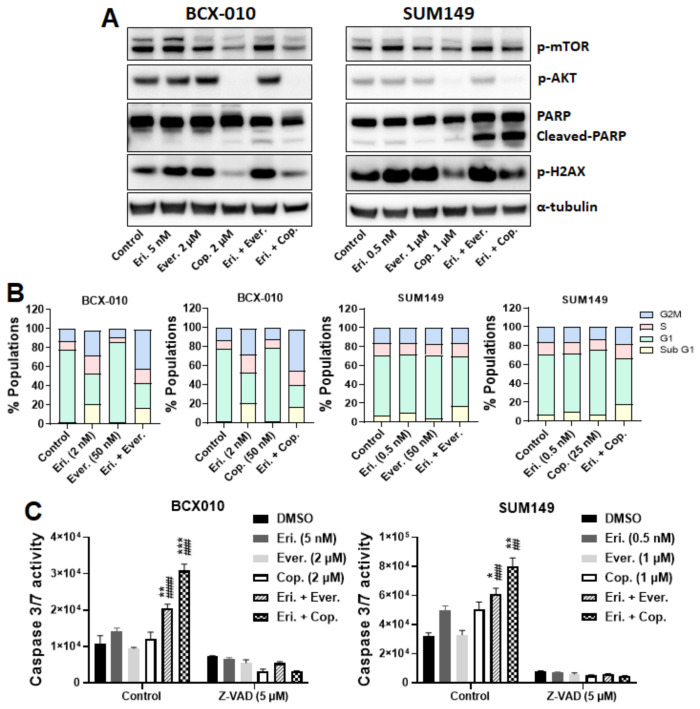
Eribulin synergizes with everolimus or copanlisib by arresting cell cycle progression and inducing apoptosis. (**A**) Cells were treated with eribulin alone, everolimus alone, copanlisib alone, eribulin plus everolimus, or eribulin plus copanlisib for 24 h. Following treatment, proteins were extracted and subjected to Western blotting analysis. Anti–α-tubulin was used as a loading control. (**B**) Cells were treated with eribulin alone, everolimus alone, copanlisib alone, eribulin plus everolimus, or eribulin plus copanlisib for 72 h. Following treatment, the cells were fixed in 70% EtOH, stained with propidium iodide, and subjected to flow cytometry analysis. (**C**) Cells were treated with DMSO or Z-VAD-FMK for 1 h and then with eribulin alone, everolimus alone, copanlisib alone, eribulin plus everolimus, or eribulin plus copanlisib for 24 h. Caspase 3/7 activity was determined using the Caspase-Glo 3/7 reagent. Data are presented as mean ± standard deviation. Statistical significance was assessed by two-tailed Student *t*-test. *, *p* < 0.05; **, *p* < 0.01; ***, *p* < 0.001. * indicates differences between the cells treated with eribulin alone and those treated with the eribulin-based combinations. ^##^, *p* < 0.01; ^###^, *p* < 0.001, ^####^, *p* < 0.0001. ^#^ indicates differences between the cells treated with a kinase inhibitor alone and those given the combination with eribulin. Eri., eribulin; Ever., everolimus; Cop., copanlisib.

To define the mechanisms of synergy between eribulin and mTOR/PI3K inhibition, we first examined the effects of combination treatments on the expression of phosphorylated Akt (pAkt), a key mediator of the PI3K/Akt/mTOR pathway [35]. Treatment with copanlisib alone reduced the expression of pAkt (Figure 5A), but no further reduction in pAkt was observed following treatment with copanlisib combined with eribulin (Figure 5A). We also examined the effects of combination treatment on the expression of phosphorylated H2AX (pH2AX), a biomarker of DNA damage [36]. Treatment with eribulin or everolimus alone increased the expression of pH2AX (Figure 5A); however, no further increase in pH2AX was observed following treatment with eribulin combined with everolimus or with copanlisib (Figure 5A). These data suggest that the synergistic effects of the combination treatment of eribulin with everolimus or with copanlisib were independent of pAkt or pH2AX.

We next examined whether the synergistic anti-proliferation effect of combination treatment was a result of cell cycle arrest and further induction of apoptosis. As shown in Figure 5B, compared to eribulin alone or everolimus alone, the combination of eribulin with everolimus increased the sub-G1 population by 7.29% and 12.96%, respectively, in SUM149 cells and increased the G2M phase by 22.62% and 40.36%, respectively, in BCX010 cells. Similarly, compared to eribulin alone or copanlisib alone, the combination of eribulin with copanlisib increased the sub-G1 population by 15.08% and 20.55%, respectively, in SUM149 cells and increased the G2M phase by 11.07% and 30.44%, respectively, in BCX-010 cells. These results suggest that the synergistic anti-proliferation effects of the combination treatment of eribulin plus everolimus or copanlisib are a result of cell cycle arrest.

To determine whether this cell cycle arrest leads to apoptotic cell death, we examined the effect of combination treatment on apoptosis induction. As shown in Figure 5A, compared to eribulin, everolimus, or copanlisib monotherapies, the combination of eribulin with everolimus or with copanlisib increased the expression of cleaved PARP in both BCX-010 and SUM149 cells, indicating the induction of apoptosis. The induction of apoptosis by combination treatments was further confirmed by the increased caspase 3/7 activity in the treated cells. As shown in Figure 5C, the combination of eribulin with everolimus significantly increased caspase 3/7 activity 1.45-fold vs. eribulin (*p* < 0.01) and 2.16-fold vs. everolimus (*p* < 0.0001) in BCX-010 cells, and 1.23-fold vs. eribulin (*p* < 0.05) and 1.84-fold vs. everolimus (*p* < 0.001) in SUM149 cells. Similarly, the combination of eribulin with copanlisib significantly increased caspase 3/7 activity 2.17-fold vs. eribulin (*p* < 0.001) and 2.53-fold vs. copanlisib (*p* < 0.001) in BCX-010 cells, and 1.61-fold vs. eribulin (*p* < 0.01) and 1.59-fold vs. copanlisib (*p* < 0.01) in SUM149 cells. To assess the potential toxicity of the combination treatment to non-tumorigenic cells, we also examined the effect of treatments on caspase 3/7 activity in MCF10A cells. Compared to eribulin, everolimus, or copanlisib monotherapies, the combination of eribulin with everolimus or copanlisib did not enhance eribulin-induced caspase 3/7 activation in MCF10A cells (Supplementary Figure S2B), suggesting that the combination does not enhance eribulin-induced apoptosis in non-tumorigenic cells. Furthermore, pre-treatment with Z-VAD-FMK (a pan-caspase inhibitor) completely inhibited the combination treatment–induced activation of caspase 3/7 in both BCX-010 and SUM149 cells (Figure 5C). Our results suggest that the synergistic anti-proliferation effect of this combination treatment is a result of the induction of apoptosis, independent of pAkt and pH2AX.

### 3.6. Combination with Inhibitors of Target Kinases Enhanced Inhibitory Activity of Eribulin against Cell Migration In Vitro

**Figure 6:**
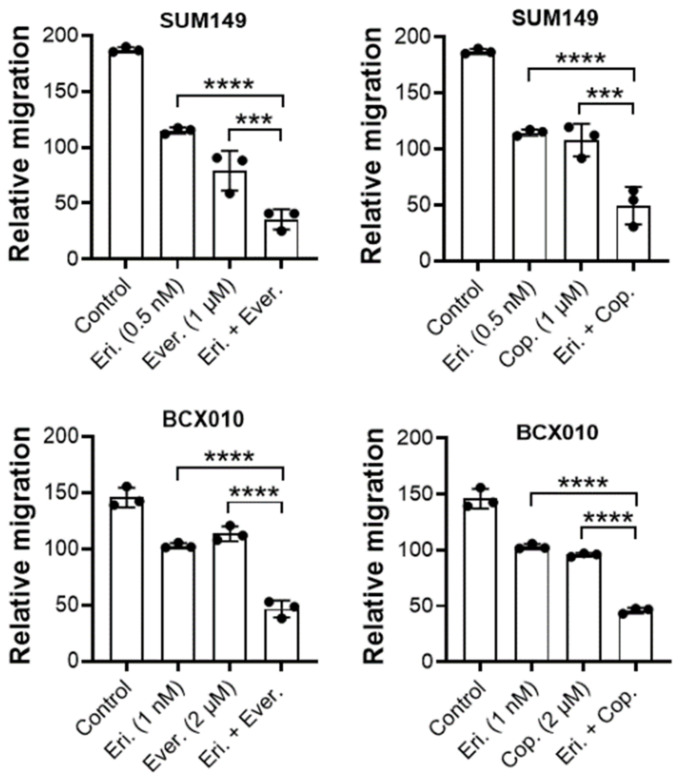
The combination of eribulin with everolimus or copanlisib is more effective than monotherapies at inhibiting TNBC cell migration. Cells were pre-treated with eribulin alone, everolimus alone, copanlisib alone, eribulin plus everolimus, or eribulin plus copanlisib for 2 h and then tested for migration using a Transwell assay in the presence of inhibitors. Data are presented as mean ± standard deviation. ***, *p* < 0.001; ****, *p* < 0.0001 by two-tailed Student *t*-test. Eri., eribulin; Ever., everolimus; Cop., copanlisib.

In addition to assessing the synergistic anti-proliferation effect of eribulin combined with the inhibitors of the PI3K/mTOR pathway, we examined the synergistic impact of the combination treatment on the motility of TNBC cells using a Transwell assay. As shown in Figure 6, compared to eribulin or everolimus monotherapies, the combination of eribulin with everolimus reduced migration by 54.61% (*p* < 0.0001) and 58.86% (*p* < 0.001), respectively, in SUM149 cells and by 69.02% and 55.00%, respectively (*p* < 0.0001 for both), in BCX-010 cells. Similarly, compared to eribulin or copanlisib monotherapies, the combination of eribulin with copanlisib reduced migration by 55.52% (*p* < 0.0001) and 52.11% (*p* < 0.001), respectively, in SUM149 cells and by 56.77% and 54.07%, respectively (*p* < 0.0001 for both), in BCX-010 cells. This result suggests that, in addition to synergistically inhibiting cell growth, the combination of eribulin with everolimus or copanlisib also inhibits the motility of TNBC cells, indicating the potential applications of this approach in controlling both tumor growth and metastasis.

## 4. Discussion

On the basis of the findings of our high-throughput kinome library RNAi screening in SUM149 cells, we extended the findings to other TNBC and TN-IBC cells and found that the combination of eribulin with inhibitors targeting the PI3K/Akt/mTOR and the MAPK/JNK pathways showed a synergistic anti-proliferation effect in those cells in vitro. Among the kinase inhibitors, the agents targeting PI3K or mTOR signaling showed the strongest synergistic effect with eribulin by inducing apoptotic cell death. These data suggest that a novel combination treatment of eribulin with everolimus or copanlisib may be promising for patients with TNBC.

PI3K is highly expressed in 40% of TNBC, and its expression is marginally higher in TNBC than in hormone receptor-expressing (HR+) breast cancer [37]. A high expression of PI3K is correlated with a larger tumor size, lymph node metastases, advanced tumor stage in TNBC [37], and unfavorable outcomes in various solid tumors [38,39,40,41]. The PI3K/mTOR pathway is also strongly activated in IBC tumors [42], and the PI3K/Akt and PI3K/mTOR pathways are crucial for IBC invasion [43,44]. In addition, *PIK3CA* is the second most commonly mutated gene after *TP53* in both TNBC (16%) and IBC (29.5%) [11,45,46]. Dysregulation of the PI3K/Akt/mTOR pathway has been implicated in genomic instability, uncontrolled proliferation, metabolic reprogramming, and resistance to therapies [14,47,48,49]. Therefore, the PI3K/Akt/mTOR pathway has been considered one of the most attractive targets for cancer treatment.

Multiple PI3K inhibitors have been developed and tested in preclinical and clinical studies. However, their efficacy as therapeutic agents is far from what is expected because of the coexistence of various mutations, compensatory feedbacks, or treatment-associated toxicity [50]. Therefore, PI3K inhibitors have been tested in combination with other drugs. For instance, the combination of buparlisib, a pan-PI3K inhibitor, with hormonal therapy showed a modest response in advanced HR+/HER2− breast cancer but displayed significant toxic effects [51]. A PI3Kα-targeting inhibitor, alpelisib, with a better toxicity profile, has been developed and is being evaluated in clinical trials in patients with HR+/HER2− metastatic breast cancer [52,53]. In a phase III clinical trial, compared to treatment with fulvestrant and placebo, treatment with alpelisib and fulvestrant increased the response rate and prolonged progression-free survival (PFS) in patients with PIK3CA-mutated HR+/HER2− advanced breast cancer (hazard ratio for progression or death, 0.65; 95% confidence interval, 0.50-0.85; *p* < 0.001) [53]. In the SOLAR-1 trial, the combination of alpelisib and fulvestrant increased the median overall survival of patients with PIK3CA-mutated HR+/HER2− advanced breast cancer compared to those treated with fulvestrant and a placebo (39.3 vs. 31.4 months; hazard ratio, 86; 95% confidence interval, 0.64–1.15; *p* = 0.15), although the analysis did not cross the prespecified boundary for statistical significance [54]. However, in the phase II/III BELLE-4 trial, patients with TNBC who were treated with buparlisib plus paclitaxel had a shorter PFS than those treated with paclitaxel alone (5.5 vs. 9.3 months; hazard ratio, 1.86; 95% confidence interval, 0.91–3.79) [55]. Moreover, patients with *PIK3CA* mutations did not show any survival benefit from the combination treatment (hazard ratio, 1.17; 95% confidence interval, 0.63–2.17) [55]. Lehmann et al. reported that *PIK3CA* mutations were highly clonal and occurred more frequently in androgen receptor–positive (AR+, 40%) TNBC vs. AR− (4%) TNBC [56], and patients with AR+ TNBC were less likely to benefit from chemotherapy. Therefore, the frequency of *PIK3CA* mutations in the TNBC molecular subtype may account for the inconsistent prognostic outcomes in clinical trials. Currently, the ongoing randomized EPIK-B3 trial is being conducted to assess the efficacy and safety of nab-paclitaxel combined with alpelisib for patients with advanced TNBC with either PIK3CA-activating mutations or PTEN loss (NCT04251533). Our results here demonstrate that the inhibition of PI3K signaling effectively enhances the anti-tumor efficacy of eribulin in vitro and ex vivo. However, the cell lines harboring *PTEN* or *PIK3CA* mutations showed a similar synergistic response to that of the wild-type cell lines, indicating that these mutations do not impact this synergistic effect.

mTOR is a key integrator of signals that control protein and lipid biosynthesis and growth factor–driven cell cycle progression [57]. mTOR was highly expressed in 44% of TNBC, and the high expression of mTOR was correlated with a high pathological prognostic TNBC stage [37]. Expression levels of mTOR were significantly higher in TNBC than in HR+ tumors, and high mTOR expression was associated with advanced tumor stage [37]. Everolimus, a derivative of rapamycin (sirolimus), inhibits mTOR activity by binding to its intracellular receptor FKBP12 to prevent the downstream signaling required for cell cycle progression and cell proliferation [58]. In the BOLERO-2 phase III study, the combination of everolimus with exemestane improved outcomes of patients with ER+/HER2− metastatic breast cancer. At an interim analysis, the combination treatment improved PFS by 4 months compared to exemestane plus a placebo (6.9 months vs. 2.8 months; hazard ratio, 0.43; 95% confidence interval, 0.35–0.54; *p* < 0.0001) [59]. At the final analysis after a median follow-up of 18 months, the median PFS was 7.8 for everolimus with exemestane vs. 3.2 months for exemestane plus placebo (hazard ratio, 0.45; 96% confidence interval 0.38–0.54; *p* < 0.0001) [60]. Based on a central assessment, the median PFS was 11.0 months for the combination vs. 4.1 months for exemestane plus placebo (*p* < 0.0001); this translates into a 62% risk reduction (hazard ratio, 0.38) [60]. The combination of everolimus with exemestane was approved by the FDA for the treatment of HR+/HER2− metastatic breast cancer. In a phase II study in patients with metastatic TNBC, the combination of everolimus and carboplatin demonstrated a clinical benefit rate of 36% and a median PFS of 3 months [61]. However, in a recently completed phase II trial in patients with TNBC, the addition of everolimus to standard neoadjuvant combination chemotherapy did not provide the expected benefit, as no significant differences were found between the everolimus-treated and nontreated groups in terms of 12-week response rates (47.8% vs. 29.6%; *p* = 0.075) and pathological complete response (30.4% vs. 25.9%; *p* = 0.76) [62]. The different clinical outcomes of combinations of everolimus with different regimens underlines the need for the careful selection of patients who may benefit from such treatments. Not all patients will respond to this combination treatment, even when patients seem to have similar molecular characteristics of subtypes of breast cancer.

The suppression of apoptosis is one of the hallmarks of tumor progression and drug resistance [63,64]. An effective approach for cancer treatment is to reactivate apoptosis in cancer cells. Thus, apoptosis has emerged as a critical endpoint for cancer treatment. Moreover, in breast cancer, PI3K/Akt/mTOR pathway activation is one of the main causes of resistance to antitumor therapies [65] and thus is an important target for improving the sensitivity of tumors to antitumor therapies. Hu et al. found that *PIK3CA* mutations triggered the sustained activation of PI3K/AKT/mTOR signaling and led to the inhibition of apoptosis in TNBC [14]. They hypothesized that agents targeting *PIK3CA* mutations and other molecules downstream of the pathway might enhance chemotherapeutic sensitivity and accelerate tumor cell apoptosis. In support of their findings, our study showed that copanlisib, which targets PI3Kα and δ isoforms, and the mTOR-targeting agent everolimus enhanced the antitumor efficacy of eribulin in both *PIK3CA*-mutated and *PIK3CA* wild-type TNBC cell lines by inducing apoptotic cell death. The results obtained using cell lines were further confirmed with fresh tumor tissue slices of TNBC cell xenografts collected from humanized mice. Fresh tumor tissues represent a more accurate in vitro model for evaluating the antitumor efficacy of drugs because of several advantages, e.g., stable gene expression profiles [66], a high degree of correlation between genetic mutations and sensitivity to targeted therapies [67], and better reproducibility [68]. These results indicate that targeting PI3K/Akt/mTOR signaling may improve the efficacy of eribulin in patients with TNBC.

However, there are several limitations of this study. These include the lack of knowledge on the association of TNBC molecular subtypes with responses to eribulin in the clinic; the correlations between (1) TNBC cell sensitivity to the combination treatment of eribulin with everolimus or copanlisib and (2) TNBC molecular subtypes, as well as (3) the genomic alterations in the PI3K/Akt/mTOR pathway; and the molecular mechanisms underlying the antitumor synergy of eribulin combined with inhibitors of the PI3K/mTOR pathway in TNBC. Such studies are critical for the translation of the combination treatment to the clinic and for the selection of patients who would benefit from such a treatment.

## 5. Conclusions

TNBC is an aggressive set of diseases, and developing optimal combination therapies for these diseases is challenging. The PI3K/mTOR/AKT pathway has been identified as a potential target for the treatment of TNBC and for overcoming resistance to chemotherapies in these diseases. Our study demonstrates that targeting PI3K/mTOR/AKT signaling with PI3K inhibitors, such as copanlisib, or with mTOR inhibitors, such as everolimus, can increase sensitivity to eribulin treatment in TNBC. Future in vivo preclinical studies, ideally using a diversity of TNBC patient-derived xenograft models, are needed to confirm the synergistic effects of eribulin combined with drugs targeting PI3K/Akt/mTOR signaling in TNBC. The findings from these studies will lay a foundation for clinical trials testing the efficacy of this strategy in patients with TNBC.

## Figures and Tables

**Table 1:** Top 135 genes showing synergistic anti-proliferation effect with eribulin (sensitivity index cutoff 0.15).

Gene Symbol	Full Gene Name	Accession #	Synergy Score	Viability (%)
*TXNDC3* (also called *NME8*)	thioredoxin domain containing 3 (spermatozoa)	NM_016616	0.37	65.64
*CMPK1*	cytidine monophosphate (UMP-CMP) kinase 1	NM_016308	0.33	39.38
*MAPK12*	mitogen-activated protein kinase 12	NM_002969	0.32	26.68
*PNCK*	pregnancy up-regulated non-ubiquitously expressed CaM kinase	NM_001039582	0.31	34.45
*TESK2*	testis-specific kinase 2	NM_007170	0.29	25.63
*TESK1*	testis-specific kinase 1	NM_006285	0.29	49.32
*NADK*	NAD kinase	NM_023018	0.29	70.86
*PANK1*	pantothenate kinase 1	NM_138316	0.29	49.30
*TAF1L*	TAF1 RNA polymerase II, TATA box binding protein (TBP)-associated factor	NM_153809	0.28	43.73
*STK36*	serine/threonine kinase 36, fused homolog (Drosophila)	NM_015690	0.28	35.39
*ALPK2*	alpha-kinase 2	NM_052947	0.28	13.26
*TRIB2*	tribbles homolog 2 (Drosophila)	NM_021643	0.27	41.05
*MVK*	mevalonate kinase	NM_000431	0.27	44.02
*SIK1*	salt-inducible kinase 1	NM_173354	0.27	32.81
*RIPK2*	receptor-interacting serine-threonine kinase 2	NM_003821	0.27	43.54
*MAP3K7IP2* (also called *TAB2*)	mitogen-activated protein kinase kinase kinase 7 interacting protein 2	NM_015093	0.26	34.00
*COL4A3BP*	collagen, type IV, alpha 3 (Goodpasture antigen) binding protein	NM_031361	0.26	40.39
*BMX*	BMX non-receptor tyrosine kinase	NM_001721	0.26	21.39
*MAK*	male germ cell-associated kinase	NM_005906	0.26	34.30
*MAP3K6*	mitogen-activated protein kinase kinase kinase 6	NM_004672	0.26	36.35
*EEF2K*	eukaryotic elongation factor-2 kinase	NM_013302	0.26	49.71
*PIK3C3*	phosphatidylinositol 3-kinase, catalytic subunit type 3	NM_002647	0.25	40.15
*TWF1*	twinfilin, actin-binding protein, homolog 1 (Drosophila)	NM_002822	0.24	23.90
*MAPK9*	mitogen-activated protein kinase 9	NM_139070	0.24	22.54
*NUAK1*	NUAK family, SNF1-like kinase, 1	NM_014840	0.24	25.75
*STK11IP*	serine/threonine kinase 11 interacting protein	NM_052902	0.24	66.15
*MAPKSP1*	MAPK scaffold protein 1	NM_021970	0.24	67.86
*PRKCI*	protein kinase C, iota	NM_002740	0.24	26.06
*CAMK2B*	calcium/calmodulin-dependent protein kinase II beta	NM_001220	0.23	29.12
*DYRK1B*	dual-specificity tyrosine-(Y)-phosphorylation regulated kinase 1B	NM_006484	0.23	72.87
*CD2*	CD2 molecule	NM_001767	0.23	35.95
*PHKG2*	phosphorylase kinase, gamma 2 (testis)	NM_000294	0.23	21.42
*PRPS1*	phosphoribosyl pyrophosphate synthetase 1	NM_002764	0.23	48.18
*AKAP12*	A kinase (PRKA) anchor protein 12	NM_005100	0.22	8.19
*PFKP*	phosphofructokinase, platelet	NM_002627	0.22	40.62
*MKNK2*	MAP kinase interacting serine/threonine kinase 2	NM_199054	0.22	58.20
*PLK2*	polo-like kinase 2	NM_006622	0.22	24.76
*DNAJC6*	DnaJ (Hsp40) homolog, subfamily C, member 6	NM_014787	0.22	31.94
*PDPK1*	3-phosphoinositide dependent protein kinase-1	NM_002613	0.22	35.37
*CDKL1*	cyclin-dependent kinase-like 1 (CDC2-related kinase)	NM_004196	0.22	15.47
*TEK*	TEK tyrosine kinase, endothelial	NM_000459	0.22	18.50
*MAP3K2*	mitogen-activated protein kinase kinase kinase 2	NM_006609	0.22	62.80
*ALPK1*	alpha-kinase 1	NM_025144	0.21	46.55
*IP6K1*	inositol hexakisphosphate kinase 1	NM_153273	0.21	34.06
*STK17A*	serine/threonine kinase 17a	NM_004760	0.21	46.33
*PLXND1*	plexin D1	NM_015103	0.21	23.60
*STK32B*	serine/threonine kinase 32B	NM_018401	0.21	26.07
*MAGI1*	membrane associated guanylate kinase, WW and PDZ domain containing 1	NM_015520	0.21	33.63
*RNASEL*	ribonuclease L (2′,5′-oligoisoadenylate synthetase-dependent)	NM_021133	0.21	41.61
*RFK*	riboflavin kinase	NM_018339	0.21	38.20
*RBKS*	ribokinase	NM_022128	0.21	49.71
*PLXNA1*	plexin A1	NM_032242	0.21	9.90
*KSR2*	kinase suppressor of ras 2	NM_173598	0.21	34.22
*FN3K*	fructosamine 3 kinase	NM_022158	0.21	18.83
*PRKCSH*	protein kinase C substrate 80K-H	NM_002743	0.20	26.97
*PIK3CA*	phosphatidylinositol-4,5-bisphosphate 3-kinase, catalytic subunit alpha	NM_006218	0.20	13.90
*PIK3C2B*	phosphoinositide-3-kinase, class 2, beta polypeptide	NM_002646	0.20	44.78
*CIB4*	calcium and integrin binding family member 4	NM_001029881	0.20	21.53
*PASK*	PAS domain containing serine/threonine kinase	NM_015148	0.20	44.89
*PAK1*	p21 protein (Cdc42/Rac)-activated kinase 1	NM_002576	0.20	30.68
*MPP2*	membrane protein, palmitoylated 2 (MAGUK p55 subfamily member 2)	NM_005374	0.20	44.16
*TAOK3*	TAO kinase 3	NM_016281	0.20	64.51
*UCK2*	uridine-cytidine kinase 2	NM_012474	0.20	64.99
*KIT*	v-kit Hardy-Zuckerman 4 feline sarcoma viral oncogene homolog	NM_000222	0.20	33.10
*CHUK*	component of inhibitor of nuclear factor kappa B kinase complex	NM_001278	0.19	25.06
*PRKD3*	protein kinase D3	NM_005813	0.19	16.40
*STK32C*	serine/threonine kinase 32C	NM_173575	0.19	35.58
*TGFBR1*	transforming growth factor, beta receptor 1	NM_004612	0.19	18.35
*TYK2*	tyrosine kinase 2	NM_003331	0.19	35.85
*ETNK2*	ethanolamine kinase 2	NM_018208	0.19	15.00
*PRKCDBP*	protein kinase C, delta binding protein	NM_145040	0.19	56.60
*MAP3K7*	mitogen-activated protein kinase kinase kinase 7	NM_003188	0.19	22.81
*MAP3K1*	mitogen-activated protein kinase kinase kinase 1	XM_042066	0.18	35.73
*DGKZ*	diacylglycerol kinase, zeta 104 kDa	NM_201532	0.18	21.25
*PCTK1*	PCTAIRE protein kinase 1	NM_033018	0.18	48.22
*NME1*	NME/NM23 nucleoside diphosphate kinase 1	NM_198175	0.18	46.25
*TLK2*	tousled-like kinase 2	NM_006852	0.18	73.65
*NME4*	NME/NM23 nucleoside diphosphate kinase 4	NM_005009	0.18	40.34
*MERTK*	c-mer proto-oncogene tyrosine kinase	NM_006343	0.18	29.48
*CAMKK2*	calcium/calmodulin-dependent protein kinase kinase 2, beta	NM_172216	0.18	23.41
*MAPK4*	mitogen-activated protein kinase 4	NM_002747	0.18	20.95
*PAK7*	p21 protein (Cdc42/Rac)-activated kinase 7	NM_020341	0.17	54.89
*ADCK2*	aarF domain containing kinase 2	NM_052853	0.17	42.05
*MAST3*	microtubule associated serine/threonine kinase 3	XM_038150	0.17	54.17
*MAP3K13*	mitogen-activated protein kinase kinase kinase 13	NM_004721	0.17	12.55
*TNNI3K*	TNNI3 interacting kinase	NM_015978	0.17	50.06
*SNX16*	sorting nexin 16	NM_022133	0.17	46.02
*PIP5K1B*	phosphatidylinositol-4-phosphate 5-kinase, type I, beta	NM_003558	0.17	26.13
*EIF2AK3*	eukaryotic translation initiation factor 2-alpha kinase 3	NM_004836	0.17	57.26
*MET*	met proto-oncogene	NM_000245	0.17	34.39
*DCK*	deoxycytidine kinase	NM_000788	0.17	22.10
*GALK1*	galactokinase 1	NM_000154	0.17	1.74
*ULK1*	unc-51-like kinase 1 (C. elegans)	XM_001133335	0.17	26.18
*ERN1*	endoplasmic reticulum to nucleus signaling 1	NM_001433	0.17	62.25
*KIAA1804*	mixed lineage kinase 4	NM_032435	0.17	20.09
*CDK5*	cyclin-dependent kinase 5	NM_004935	0.17	17.19
*TWF2*	twinfilin actin-binding protein 2	NM_007284	0.16	34.93
*MAPK7*	mitogen-activated protein kinase 7	NM_139034	0.16	49.65
*BMPR1A*	bone morphogenetic protein receptor, type IA	NM_004329	0.16	11.85
*CDK2*	cyclin-dependent kinase 2	NM_001798	0.16	43.72
*PKMYT1*	protein kinase, membrane associated tyrosine/threonine 1	NM_004203	0.16	11.64
*LTK*	leukocyte receptor tyrosine kinase	NM_206961	0.16	15.55
*CDK10*	cyclin-dependent kinase 10	NM_003674	0.16	12.80
*FGFRL1*	fibroblast growth factor receptor-like 1	NM_021923	0.16	24.80
*RPS6KA6*	ribosomal protein S6 kinase, 90 kDa, polypeptide 6	NM_014496	0.16	24.56
*GNE*	glucosamine (UDP-N-acetyl)-2-epimerase/N-acetylmannosamine kinase	NM_005476	0.16	31.57
*MARK1*	MAP/microtubule affinity-regulating kinase 1	NM_018650	0.16	39.49
*PLK3*	polo like kinase 3	NM_004073	0.16	24.31
*NRK*	Nik related kinase	NM_198465	0.16	17.66
*SH3BP5L*	SH3-binding domain protein 5-like	NM_030645	0.16	34.86
*UHMK1*	U2AF homology motif (UHM) kinase 1	NM_175866	0.16	48.37
*AK5*	adenylate kinase 5	NM_012093	0.16	28.30
*MAP3K9*	mitogen-activated protein kinase kinase kinase 9	NM_033141	0.16	29.05
*CKB*	creatine kinase B	NM_001823	0.16	39.76
*PIK3CB*	phosphoinositide-3-kinase, catalytic, beta polypeptide	NM_006219	0.16	33.44
*CDC42BPG*	CDC42 binding protein kinase gamma (DMPK-like)	NM_017525	0.16	11.56
*GUK1*	guanylate kinase 1	NM_000858	0.15	29.61
*TRIB1*	tribbles pseudokinase 1	NM_025195	0.15	20.63
*MPP3*	membrane protein, palmitoylated 3 (MAGUK p55 subfamily member 3)	NM_001932	0.15	35.52
*DAK*	dihydroxyacetone kinase 2 homolog	NM_015533	0.15	50.20
*TEC*	tec protein tyrosine kinase	NM_003215	0.15	14.44
*CHKA*	choline kinase alpha	NM_212469	0.15	41.37
*MAP3K3*	mitogen-activated protein kinase kinase kinase 3	NM_203351	0.15	33.43
*NRGN*	neurogranin (protein kinase C substrate, RC3)	NM_006176	0.15	45.45
*FUK*	fucokinase	NM_145059	0.15	23.89
*FLT1*	fms-related tyrosine kinase 1	NM_002019	0.15	19.52
*PIP4K2B*	phosphatidylinositol-5-phosphate 4-kinase type 2 beta	NM_003559	0.15	29.44
*NME9*	NME/NM23 family member 9	NM_178130	0.15	35.70
*ROR1*	receptor tyrosine kinase like orphan receptor 1	NM_001083592	0.15	48.46
*MAP2K2*	mitogen-activated protein kinase kinase 2	NM_030662	0.15	63.34
*MAP4K3*	mitogen-activated protein kinase kinase kinase kinase 3	NM_003618	0.15	54.72
*PTK6*	protein tyrosine kinase 6	NM_005975	0.15	21.38
*MAP3K19*	mitogen-activated protein kinase kinase kinase 19	NM_025052	0.15	52.52
*DCAKD*	dephospho-CoA kinase domain containing	NM_024819	0.15	45.86
*PRKCG*	protein kinase C gamma	NM_002739	0.15	33.51

## Data Availability

Not applicable.

## Supplementary Materials

The following are available online at https://www.mdpi.com/article/10.3390/biomedicines11030735/s1, Supplementary method: clonogenic assay, Figure S1: combination of eribulin with everolimus or copanlisib was more effective than monotherapies at inhibiting TNBC cell growth, Figure S2: combination of eribulin with everolimus or copanlisib had no synergistic effect on MCF10A cell growth and did not enhance eribulin-induced apoptosis.

## Abbreviations

ER—estrogen receptor; FDA—U.S. Food and Drug Administration; HER2—human epidermal growth factor receptor 2; HR—hormone receptor; PARP—poly(ADP-ribose) polymerase; PI3K—phosphatidylinositol-3-kinase; PR—progesterone receptor; TNBC—triple-negative breast cancer; TN-IBC—triple-negative inflammatory breast cancer.
