# Supplementary material for: Identification of Kinase Targets for Enhancing the Antitumor Activity of Eribulin in Triple-Negative Breast Cell Lines
Source: Biomedicines. 2023 Feb 28;11(3):735. doi: 10.3390/biomedicines11030735 (PMC10045293; doi:10.3390/biomedicines11030735)
Supplement: Supplementary file 1 [file biomedicines-11-00735-s001.zip › biomedicines-2242371-supplementary.pdf]

Identification of Kinase Targets for Enhancing the Antitumor Activity of Eribulin in Triple-Negative Breast Cell Lines

Supplementary Materials and Methods

Clonogenic assay

A clonogenic assay was performed to determine the effect of treatments on cell growth. MCF10A cells (500 cells/well) were seeded in six-well plates and cultured overnight. The cells were treated the next morning with eribulin alone, everolimus alone, copanlisib alone, eribulin plus everolimus, or eribulin plus copanlisib for 5-7 days at 37 °C. After treatment, the colonies were stained with MTT (3-[4,5-dimethylthiazol-2-yl]-2,5-diphenyltetrazolium bromide; #M5655; Sigma-Aldrich, St. Louis, MO, USA), and colonies > 80 µm in diameter were counted using the GelCount system (Oxford Optronix Ltd, Milton Park, Abingdon, UK).

Supplementary Results

Supplementary Figure S1

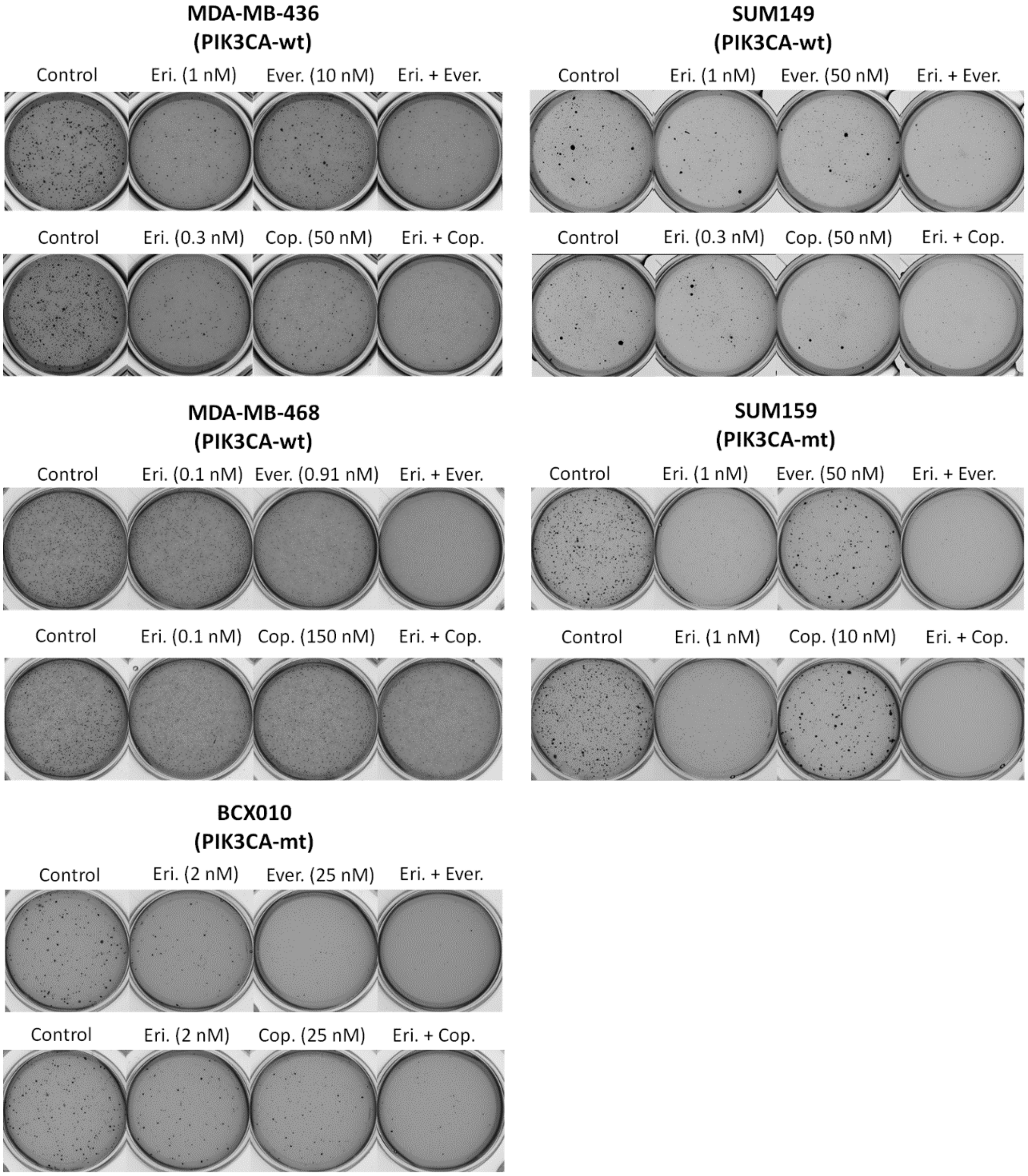

Supplementary Figure S1. Combination of eribulin with everolimus or copanlisib was more effective than monotherapies at inhibiting TNBC cell growth. The synergistic effects of combination treatment were determined using a soft agar colony formation assay at weeks 3 or 4 following treatment with eribulin alone, everolimus alone, copanlisib alone, eribulin plus everolimus, or eribulin plus copanlisib. Eri., eribulin; Ever., everolimus; Cop., copanlisib; mt, mutated; and wt, wild-type.

Supplementary Figure S2

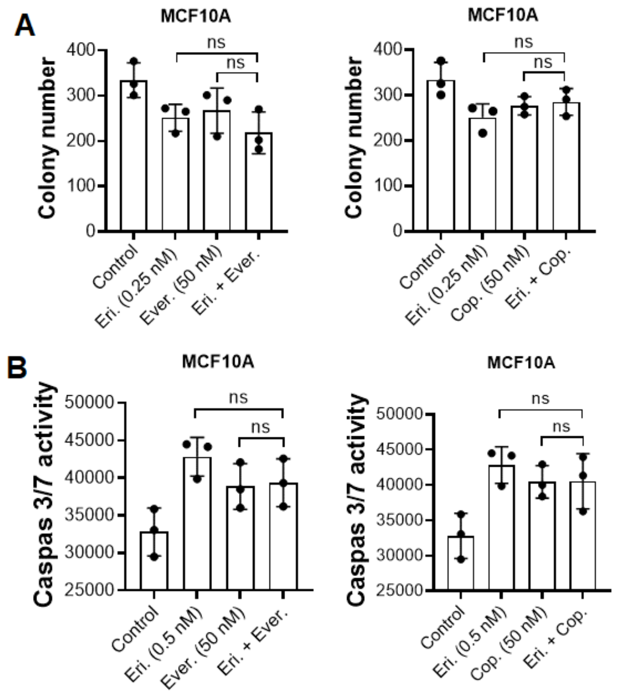

Supplementary Figure S2. Combination of eribulin with everolimus or copanlisib had no synergistic effect on MCF10A cell growth and did not enhance eribulin-induced apoptosis. (**A**) The synergistic effects of combination treatment were determined using a clonogenic assay at days 5-7 following treatment with eribulin alone, everolimus alone, copanlisib alone, eribulin plus everolimus, or eribulin plus copanlisib. (**B**) The apoptosis induction following treatment was determined by measuring caspase 3/7 activity. Cells were treated with eribulin alone, everolimus alone, copanlisib alone, eribulin plus everolimus, or eribulin plus copanlisib for 12 h, and then caspase 3/7 activity was determined using Caspase-Glo 3/7 reagent. Eri., eribulin; Ever., everolimus; Cop., copanlisib; and ns, not significant.
